# Current attitudes of primary care providers toward albuminuria testing in patients with diabetes in the United States

**DOI:** 10.1016/j.pmedr.2026.103581

**Published:** 2026-07-18

**Authors:** Yoshihisa Miyamoto, Fang Xu, Ibrahim Zaganjor, Meda E. Pavkov, Alain K. Koyama, Stephen Onufrak

**Affiliations:** Division of Diabetes Translation, National Center for Chronic Disease Prevention and Health Promotion, CDC, Atlanta, GA, USA

**Keywords:** Chronic kidney disease, Screening, Diabetes, Clinical care, Clinical guideline compliance, Barriers and facilitators

## Abstract

**Objective:**

To evaluate primary care providers' attitudes and practices regarding albuminuria testing in patients with type 2 diabetes in the United States.

**Methods:**

Using a web-based opt-in panel of providers (Porter Novelli DocStyles survey, Fall 2024), we conducted a cross-sectional study to assess attitudes toward albuminuria testing, adherence to guidelines, and reported barriers. Associations were estimated using multivariable Poisson regression.

**Results:**

Among 1236 providers who see at least one patient with diabetes weekly, 90.0% supported albuminuria testing and 83.3% agreed that annual testing helps with diabetes prognosis. Adherence to recommended urine testing—defined as correct test type, concurrent urine creatinine measurement, and recommended testing frequency—ranged from 52% to 68%. Lower adherence was associated with inpatient practice, being a nurse practitioner or physician assistant, lack of a urinalysis reminder system, perceived guideline ambiguity or time constraints, and lower diabetes patient volume.

**Conclusions:**

Despite most providers supporting urine testing for patients with diabetes, significant gaps between knowledge and practice were identified. Clinical decision support tools and clearer recommendations may help close the gaps between the guideline recommendations and practice.

## 1 Introduction

Chronic kidney disease (CKD) is a common complication among adults with diabetes, affecting approximately 38% of individuals with diabetes over their lifetime (de Boer et al., 2022). CKD is generally asymptomatic and diagnosed based on measurement of serum creatinine for estimating glomerular filtration rate (eGFR) and urine albumin concentration (Levey et al., 2022). Early detection of CKD is critical for preventing disease progression through interventions such as angiotensin-converting enzyme inhibitors (ACEi) or angiotensin receptor blockers (ARBs) (Chen et al., 2019) and newer treatments, including sodium–glucose cotransporter-2 inhibitors (SGLT2i) (American Diabetes Association Professional Practice Committee, 2024). However, awareness of CKD remains very low, with nine out of ten affected adults unaware of their condition (Chu et al., 2021). This lack of awareness may stem from the underutilization of albuminuria testing (Chu et al., 2023), an essential tool for identifying CKD in its early stages, as recommended in clinical guidelines (American Diabetes Association Professional Practice Committee, 2024).

Albuminuria is a strong predictor not only for kidney failure (Liu et al., 2024; Tangri et al., 2016) but also for cardiovascular diseases (Barzilay et al., 2024), the most frequently lethal complications of CKD. Increases in albuminuria can be used as a surrogate endpoint for predicting CKD progression (Heerspink et al., 2015, Heerspink et al., 2019), and treatments that reduce albuminuria slow or prevent the progression of CKD^12^. Emerging treatments such as finerenone—a nonsteroidal mineralocorticoid receptor antagonist (Bakris et al., 2023)—and glucagon-like peptide-1 receptor agonists (GLP-1 RAs) have shown promise in reducing albuminuria and improving kidney and cardiovascular outcomes in patients with diabetes-associated CKD.

Despite guidelines from organizations such as the American Diabetes Association, which recommend annual urinary albumin testing for all individuals with type 2 diabetes regardless of treatment status (American Diabetes Association Professional Practice Committee, 2024), implementation rates remain suboptimal. Barriers to testing include time constraints during primary care visits, logistical challenges in outpatient settings, and both clinician- and patient-related factors such as knowledge gaps or reluctance to undergo urine tests (Neale et al., 2020; Groehl et al., 2023; Stempniewicz et al., 2021). Recent multidisciplinary guidance further reinforces the importance of albuminuria testing in diabetes. The 2024 Kidney Disease: Improving Global Outcomes clinical practice guideline recommends combined assessment of estimated glomerular filtration rate (eGFR) and albuminuria for evaluation and risk stratification of chronic kidney disease, and the American Heart Association PREVENT equations incorporate albuminuria into cardiovascular-kidney-metabolic risk assessment (Kidney Disease: Improving Global Outcomes (KDIGO) CKD Work Group, 2024; Khan et al., 2024).

While administrative claims data provide insights into testing patterns, they fail to capture underlying barriers. Furthermore, most previous surveys of clinicians were conducted before the broad clinical introduction of SGLT2 inhibitors and GLP-1 RAs. Given that the CKD management paradigm has been shifted, updated data are needed to reflect the current clinical landscape. To bridge this gap, we surveyed primary care providers using the DocStyles web-based platform to explore attitudes and perceived obstacles regarding albuminuria testing. Given that PCPs help manage the clinical needs of patients with diabetes in the United States, understanding their perspectives is crucial for enhancing CKD detection and management strategies.

## 2 Methods

### 2.1 Study design and population

We conducted a cross-sectional study using data from the Fall 2024 Porter Novelli DocStyles survey, a web-based survey of U.S. healthcare providers (Porter Novelli, 2024). From August 29 to September 27, 2024, the survey was administered by M3 Global Research for Porter Novelli. Respondents were eligible if they practiced in the United States, actively saw patients, worked in an individual, group, or hospital practice, and had been in practice for at least three years. Invitations were sent sequentially to high responders, followed by medium and low responders, with random sampling within each response tier. The Centers for Disease Control and Prevention (CDC) has a licensing agreement with Porter Novelli for DocStyles 2024, which specifically included questions related to diabetes and CKD.

To meet quota targets, 2990 health professionals were invited to participate in Fall 2024 DocStyles, and 1753 respondents who practiced in the United States completed the survey, yielding an overall response rate of 59%. The sample of primary care providers included 1001 primary care physicians, 250 obstetricians or gynecologists, 252 pediatricians, 109 nurse practitioners, and 141 physician assistants. Response rates by specialty were 57% for primary care physicians, 70% for pediatricians, 56% for obstetricians/gynecologists, 51% for nurse practitioners, and 64% for physician assistants. Healthcare providers who reported seeing an average of fewer than one adult patient with diabetes weekly and all pediatricians and obstetricians/gynecologists were not included in the present study. This resulted in a final study sample of 1236 primary care providers.

### 2.2 Measures

The survey assessed provider attitudes and practices regarding albuminuria testing in patients with diabetes, perceived barriers to testing, and use of clinical decision support tools. Provider characteristics collected included age, sex, race, Hispanic ethnicity, medical specialty, years in practice, weekly patient volume, work setting, and teaching hospital status. For the variables in this study, there were no missing data. We evaluated two outcomes related to agreement or disagreement with recommended guidelines for albuminuria testing:

• Agreement with the recommendation to test for albuminuria in patients with diabetes
• Agreement that albuminuria testing once a year is helpful to predict the prognosis of diabetes

We evaluated five outcomes related to adherence to guideline-recommended albuminuria testing:

• Quantitative albuminuria testing (use of a quantitative method to detect albuminuria).
• Quantitative albuminuria testing with concurrent urine creatinine measurement (ordering urine creatinine together with albumin to estimate the urinary albumin/creatinine ratio.
• Annual albuminuria testing for patients with eGFR ≥60 mL/min/1.73 m^2^.
• Annual albuminuria testing for patients with eGFR <60 mL/min/1.73 m^2^.
• All recommended guideline components (meeting all criteria above).

### 2.3 Statistical analysis

The percentage of healthcare providers who agreed and did not agree that annual albuminuria testing is helpful to predict the prognosis of diabetes was compared according to provider characteristics using χ^2^ tests. Associations between provider characteristics, perceived barriers, and adherence were assessed using multivariable Poisson regression models. The multivariable models included the following covariates: provider age group (<50, ≥50 years), sex, race (White, Black/African American, Asian, and other), Hispanic ethnicity, medical specialty (family practitioner, internist, nurse practitioner/physician assistant), years in clinical practice (<15, ≥15 years), weekly total patient volume (1–50, 51–100, ≥101), weekly diabetes patient volume (1–9, 10–19, ≥20), primary clinical work setting (individual outpatient, group outpatient, inpatient), employment at a teaching hospital (yes/no), perceived barriers (guideline ambiguity, time limitations, patient refusal, operational challenges, and patient financial burden), and use of a urinalysis reminder system.

Only variables that were statistically significant in multivariable analyses are presented in the main results table describing the fully adjusted models; non-significant variables are not shown but were retained in the models. The full survey items and outcome definitions are provided in Supplemental Table 1.

All analyses were performed using R version 4.3.2. The data did not include identifiable information, and this activity was reviewed by CDC and was conducted in accordance with applicable federal law and CDC policy.

## 3 Results

Among 1236 health care providers who see at least one patient with diabetes weekly, 39% were internists, 42% were family practitioners, and 19% were nurse practitioners and physician assistants (Table 1). Most providers practiced in group outpatient settings (68%), were under 50 years old (61%), were male (55%) and White (64%). Overall, 83% of providers agreed that annual testing is helpful for predicting diabetes prognosis, with agreement differing significantly by medical specialty, age, sex, race, teaching hospital employment, and years in practice (Table 1).

**Table 1:** Agreement that annual albuminuria testing is helpful for predicting diabetes prognosis among primary care providers in the United States, Porter Novelli DocStyles survey, 2024.

Provider Characteristic	Category (% in Study Sample)	Do Not Agree (%)	Agree (%)	*p*-value
Overall (*N* = 1236)		16.7% (*N* = 207)	83.3% (*N* = 1029)	N/A
Medical Specialty				<0.01
	Internist (39.0%)	11.4%	88.6%	
	Family Practitioner (42.0%)	15.7%	84.3%	
	Nurse practitioners and physician assistants (19.0%)	29.8%	70.2%	
Age				<0.01
	<50 years (61.0%)	19.2%	80.8%	
	≥50 years (39.0%)	12.9%	87.1%	
Sex				<0.01
	Female (45.0%)	20.3%	79.7%	
	Male (55.0%)	13.9%	86.1%	
Race				<0.01
	White (64.0%)	19.3%	80.7%	
	Black/African American (4.0%)	16.0%	84.0%	
	Asian (23.0%)	9.2%	90.8%	
	Other (8.6%)	17.9%	82.1%	
Hispanic Origin				0.90
	Yes (7.0%)	17.2%	82.8%	
	No (93.0%)	16.7%	83.3%	
Work Setting				0.41
	Individual outpatient (19.0%)	16.4%	83.6%	
	Group outpatient (68.0%)	16.2%	83.8%	
	Inpatient (13.0%)	20.4%	79.6%	
Teaching Hospital				0.02
	Yes (42.0%)	13.7%	86.3%	
	No (58.0%)	18.9%	81.1%	
Years in Practice				<0.01
	<15 years (53.0%)	19.6%	80.4%	
	≥15 years (47.0%)	13.5%	86.5%	
Weekly Patient Volume				0.6
	1–50 (18.0%)	18.9%	81.1%	
	51–100 (55.0%)	16.2%	83.8%	
	≥101 (27.0%)	16.5%	83.5%	
Weekly Diabetes Patient Volume				0.17
	1–9 (13.0%)	21.9%	78.1%	
	10–19 (31.0%)	15.6%	84.4%	
	≥20 (56.0%)	16.2%	83.8%	

“Do not agree” includes those who responded “neither agree nor disagree,” “disagree,” or “strongly disagree” to the statement that annual albuminuria testing is helpful to predict the prognosis of diabetes (Question 3, Supplemental Table).

*P*-values were calculated using chi-square tests.

Overall, 90% of providers agreed with the recommendation to test for albuminuria in patients with diabetes (data not shown). However, only 68% adhered to quantitative albuminuria testing, 52% consistently ordered concurrent urine creatinine measurement, and 64 and 65% performed annual albuminuria testing for patients with eGFR ≥60 or < 60 mL/min/1.73 m^2^, respectively (Table 2). Adherence to all recommended guideline components was 50%.

**Table 2:** Associations of provider characteristics and perceived barriers with adherence to albuminuria testing recommendations among primary care providers in the United States, Porter Novelli DocStyles survey, 2024 (N = 1236).

Provider Characteristics	Adherence to Quantitative Albuminuria Testing^1^ PR (95% CI)	Adherence to Quantitative Albuminuria + Concurrent Urine Creatinine^2^ PR (95% CI)	Adherence to Annual Albuminuria Testing for eGFR ≥ 60^3^ PR (95% CI)	Adherence to Annual Albuminuria Testing for eGFR < 60^4^ PR (95% CI)	Adherence to All Guideline Components^5^ PR (95% CI)
**Overall Prevalence of Adherence**	68%	52%	64%	65%	50%

*Weekly diabetes patient volume*					
1–9 (ref)	1.00	1.00	1.00	1.00	1.00
10–19	1.24 (1.06, 1.45)	1.30 (1.04, 1.62)	1.25 (1.06, 1.48)	1.24 (1.06, 1.47)	1.27 (1.02, 1.59)
≥20	1.26 (1.08, 1.47)	1.50 (1.21, 1.86)	1.31 (1.11, 1.55)	1.31 (1.12, 1.55)	1.48 (1.19, 1.84)

*Medical specialty*					
Internist (ref)	1.00	1.00	1.00	1.00	1.00
Family Practitioner	0.94 (0.87, 1.01)	0.89 (0.80, 0.99)	0.97 (0.89, 1.05)	0.94 (0.86, 1.01)	0.90 (0.81, 1.01)
Nurse practitioners and physician assistants	0.63 (0.54, 0.74)	0.55 (0.44, 0.68)	0.63 (0.53, 0.74)	0.57 (0.48, 0.68)	0.52 (0.41, 0.65)

*Perceived barrier: Guideline ambiguity*					
Agree/Strongly agree	0.80 (0.69, 0.93)	0.66 (0.53, 0.82)	0.75 (0.63, 0.89)	0.77 (0.66, 0.90)	0.64 (0.50, 0.80)
Neither agree nor disagree/Disagree/Strongly disagree (ref))	1.00	1.00	1.00	1.00	1.00

*Use of urinalysis reminder system*					
Yes	1.20 (1.11, 1.29)	1.23 (1.10, 1.37)	1.23 (1.13, 1.34)	1.21 (1.12, 1.31)	1.27 (1.14, 1.42)
No (ref)	1.00	1.00	1.00	1.00	1.00

*Primary clinical work setting*					
Individual outpatient (ref)	1.00	1.00	1.00	1.00	1.00
Group/clinic	1.09 (0.98, 1.21)	1.07 (0.93, 1.23)	1.06 (0.95, 1.18)	1.06 (0.96, 1.18)	1.05 (0.91, 1.22)
Inpatient	0.83 (0.69, 1.00)	0.65 (0.50, 0.84)	0.74 (0.60, 0.92)	0.78 (0.64, 0.95)	0.63 (0.48, 0.83)

*Perceived barrier: Time limitations*					
Agree/Strongly agree	0.90 (0.78, 1.04)	0.78 (0.64, 0.96)	0.75 (0.62, 0.89)	0.85 (0.73, 1.00)	0.69 (0.54, 0.86)
Neither agree nor disagree/Disagree/Strongly disagree (ref)	1.00	1.00	1.00	1.00	1.00

*Perceived barrier: Patient refusal*					
Agree/Strongly agree	1.13 (1.01, 1.28)	1.21 (1.03, 1.41)	1.09 (0.95, 1.25)	1.14 (1.00, 1.29)	1.21 (1.02, 1.43)
Neither agree nor disagree/Disagree/Strongly disagree (ref)	1.00	1.00	1.00	1.00	1.00

PR = Prevalence Ratio (from multivariable analysis).

Ref = Referent groups.

CI = Confidence Interval.

Multivariable models included provider age group, sex, race, Hispanic ethnicity, medical specialty, years in clinical practice, weekly total patient volume, weekly diabetes patient volume, primary clinical work setting, employment at a teaching hospital, all perceived barriers (guideline ambiguity, time limitations, patient refusal, operational challenges, and patient financial burden), and use of a urinalysis reminder system.

Age, sex, race and ethnicity, years in practice, weekly total patient volume, teaching hospital status, and some barrier variables (operational/logistical challenges, financial burden) were included in the model but are not presented in the table as they were not significant for any outcomes in multivariable analysis.

1 **Adherence to Quantitative Albuminuria Testing:** Defined as using a quantitative method (not dipstick or non-specific proteinuria) to detect albuminuria in patients with diabetes.

2 **Adherence to Quantitative Albuminuria + Concurrent Urine Creatinine:** Defined as always ordering a urine creatinine test concurrently with quantitative albuminuria to estimate the urine albumin-creatinine ratio (UACR).

3 **Adherence to Annual Albuminuria Testing for eGFR ≥** **60:** Defined as performing annual quantitative albuminuria testing for patients with diabetes whose estimated glomerular filtration rate (eGFR) is ≥60 mL/min/1.73 m^2^.

4 **Adherence to Annual Albuminuria Testing for eGFR <** **60:** Defined as performing annual quantitative albuminuria testing for patients with diabetes whose eGFR is <60 mL/min/1.73 m^2^.

5 **Adherence to All Guideline Components:** Defined as adherence to all of the above components: quantitative albuminuria testing, concurrent urine creatinine measurement, and annual testing for both eGFR categories.

Providers seeing more patients with diabetes weekly (≥10) had higher adherence across all five guideline outcomes (adjusted prevalence ratios [PRs] = 1.24–1.30 for 10, 19 diabetes patients weekly and 1.26–1.50 for ≥ 20 weekly compared to those seeing 1–9 patients/week; Table 2). nurse practitioners/physician assistants had significantly lower adherence compared to internists (PRs 0.52–0.63). Working in inpatient settings was associated with reduced adherence (PRs 0.63–0.83) compared with individual outpatient practice.

Perceived guideline ambiguity and time limitations were consistently associated with lower adherence (PRs 0.64–0.80 and 0.69–0.90, respectively; Table 2). Use of a urinalysis reminder system was associated with improved adherence (PRs 1.20–1.27). Interestingly, perceiving patient refusal as a barrier was associated with higher adherence in several outcomes (PRs 1.13–1.21).

Prevalence of reported barriers to albuminuria testing were: patient financial burden (19.8%; 95% CI: 17.6, 22.0), operational challenges (16.7%; 95% CI: 14.6, 18.8), time constraints (15.5%; 95% CI: 13.5, 17.5), unclear guidelines (15.3%; 95% CI: 13.3, 17.3), and patient refusal (12.3%; 95% CI: 10.5, 14.1) (Data not shown).

## 4 Discussion

This study using the DocStyles survey highlights the adherence of primary care providers to albuminuria testing guidelines for patients with diabetes and identifies factors potentially influencing adherence. Although 90% of providers agreed with the recommendation to test albuminuria in patients with diabetes, only half of providers adhered to all components of albuminuria testing. This suggests that the main barrier may not be disagreement with recommendations, but rather the practical challenges associated with implementation. Factors such as the use of reminder systems and certain provider demographic factors were associated with higher adherence levels while perceived guideline ambiguity was associated with lower adherence.

The use of quantitative albuminuria as a screening method for patients with diabetes was higher compared to findings from previous studies. For example, a meta-analysis by a global consortium showed the urine albumin-to-creatinine ratio screening rate was 35.1% (12.3–74.5%) in participants with diabetes in the clinical cohorts (Shin et al., 2021). More recent data involving over 513,000 adults with type 2 diabetes in primary care settings showed the one-year median testing rate for urine albumin-to-creatinine ratio by site was 52.9%, with considerable variability across organizations or sites (Stempniewicz et al., 2021). Ultimately, the fact that only half of providers adhered to all recommended albuminuria testing components indicates significant room for improvement in comprehensive guideline adherence.

The gap between agreement with clinical guidelines and adherence to recommended testing practices highlights a disconnect between provider knowledge/awareness and implementation in routine care. To our knowledge, this is the first large-scale survey to specifically examine attitudes and barriers of primary care providers' adherence to albuminuria testing for patients with diabetes in the modern clinical era, characterized by the availability of newer agents such as SGLT2i and GLP1-RA. A 2023 review study did not identify newer studies after 2021 specifically examining this issue among patients with diabetes (Williamson et al., 2023). Confusion over the variety of available urine tests (e.g., microalbuminuria, albuminuria, proteinuria, urine albumin-to-creatinine ratio, and urine protein-creatinine ratio) may in part contribute to suboptimal adherence (Oude Engberink et al., 2025). Findings that perceived ambiguity in guidelines was associated with lower adherence suggest the potential benefit of clearer, more actionable recommendations to bridge the knowledge-practice gap.

Lower agreement or adherence to the guideline for NP/PA highlights opportunities to increase NP/PA adherence. These findings were consistent with a previous Medicare study (Buerhaus et al., 2018) where beneficiaries cared for by NP had fewer hospital admissions, readmissions, and inappropriate emergency department visits but those receiving care from medical doctors were more likely to receive chronic disease management, including medical attention for nephropathy. Other research found that patients managed by healthcare teams including NP/PA received more guideline-recommended diabetes care overall (Stempniewicz et al., 2021). Taken together, this suggests the complementary role of nurse practitioners and physician assistants in enhancing process metrics and care coordination.

Current quality-measure initiatives may also shape clinician behavior. The Kidney Health Evaluation for Patients With Diabetes measure emphasizes both eGFR assessment and urine albumin-to-creatinine ratio testing and is relevant to current U.S. quality programs, including the Healthcare Effectiveness Data and Information Set and the Merit-based Incentive Payment System. This measure has been associated with improved detection and management of chronic kidney disease (Ferre et al., 2023). Because our survey was conducted in Fall 2024, responses may reflect a period of transition in awareness and implementation of these newer quality measures. Continued uptake of such measures may improve albuminuria testing in future practice.

There are several limitations to this study. First, the DocStyles panel and its data may not accurately represent all primary care providers, as participation in the survey was voluntary, potentially introducing selection bias of providers who may be more adherent than the general population of providers. Second, the survey is self-reported, which is subject to social desirability bias, as respondents may overestimate behaviors they perceive as desirable. Third, since the survey questioned providers about adherence, the results may not be directly comparable with studies performed at the patient level. Finally, the study is cross-sectional in design, which does not allow assumptions of causality in the reported associations.

## 5 Conclusions

Despite most primary care providers supporting urine testing for patients with diabetes, significant gaps between knowledge and practice were identified. Clinical decision support tools and clearer recommendations may help close the gaps between the guideline recommendations and practice.

## Declaration of generative AI and AI-assisted technologies in the manuscript preparation process

During the preparation of this work, the authors used ChatGPT for editing of the manuscript draft for clarity and brevity and formatting of tables. The authors reviewed and edited the output as needed and take full responsibility for the content of the published article.

## AI Statement

AI was used for formatting and editing of the manuscript.

## CRediT authorship contribution statement

**Yoshihisa Miyamoto:** Writing – review & editing, Writing – original draft, Visualization, Methodology, Investigation, Formal analysis, Data curation, Conceptualization. **Fang Xu:** Writing – review & editing, Methodology, Conceptualization. **Ibrahim Zaganjor:** Writing – review & editing, Methodology, Conceptualization. **Meda E. Pavkov:** Writing – review & editing, Supervision, Project administration, Conceptualization. **Alain K. Koyama:** Writing – review & editing, Visualization, Methodology. **Stephen Onufrak:** Writing – review & editing, Visualization, Supervision, Project administration, Methodology, Conceptualization.

## Disclaimer

The findings and conclusions in this report are those of the authors and do not necessarily represent the official position of the Centers for Disease Control and Prevention.

## Funding

This work was performed as part of the authors' regular duties as employees of the Federal Government. No additional funding was obtained.

## Declaration of competing interest

The authors declare that they have no known competing financial interests or personal relationships that could have appeared to influence the work reported in this paper.

## Appendix A Supplementary data

Supplemental Table. Survey items and outcome definitions for albuminuria testing among primary care providers in the United States, Porter Novelli DocStyles survey, Fall 2024.
