# Supplementary material for: Current attitudes of primary care providers toward albuminuria testing in patients with diabetes in the United States
Source: Prev Med Rep. 2026 Jul 18;69:103581. doi: 10.1016/j.pmedr.2026.103581 (PMC13427520; doi:10.1016/j.pmedr.2026.103581)
Supplement: Supplementary file 1 — Supplemental Table. Survey items and outcome definitions for albuminuria testing among primary care providers in the United States, Porter Novelli DocStyles survey, Fall 2024. [file mmc1.docx]

**Supplemental Table. Survey items and outcome definitions for albuminuria testing among primary care providers in the United States, Porter Novelli DocStyles survey, Fall 2024.**

|  | **Survey Item** | **Response Options** | **Use in Analysis / Outcome Definition** |
| --- | --- | --- | --- |
|  | On average, how many adult patients with diabetes do you see in a typical week? | 0; 1–9; 10–19; 20 or more | Used to define eligible sample and categorize weekly diabetes patient volume |
|  | Do you agree or disagree with the recommendation to test for albuminuria in patients with diabetes? | Strongly agree; Agree; Neither agree nor disagree; Disagree; Strongly disagree | Used to assess provider agreement with guidelines |
|  | Do you agree or disagree that albuminuria testing once a year is helpful to predict the prognosis of diabetes? | Strongly agree; Agree; Neither agree nor disagree; Disagree; Strongly disagree | Used to assess provider agreement with annual testing for prognosis |
|  | In your practice, which approach do you use to detect albuminuria? | Dipstick urine test; Quantitative albuminuria; Quantitative proteinuria; Other tests; Never order tests | **Outcome 1:** Adherence to quantitative albuminuria testing |
|  | How often do you order a urine creatinine test concurrently with quantitative albuminuria to estimate urine albumin-to-creatinine ratio? | Always; Often; Sometimes; Rarely; Never; Not sure | **Outcome 2:** Adherence to quantitative albuminuria + concurrent urine creatinine (defined as "Always") |
|  | How often do you order an albuminuria test for a patient with diabetes whose eGFR is ≥60 ml/min/1.73㎡? | More than once per year; Once a year; Once every 2–3 years; Once every 4+ years; Never | **Outcome 3:** Adherence to annual albuminuria testing for eGFR ≥60 (defined as "Once a year" or more) |
|  | How often do you order an albuminuria test for a patient with diabetes whose eGFR is <60 ml/min/1.73㎡? | More than once per year; Once a year; Once every 2–3 years; Once every 4+ years; Never | **Outcome 4:** Adherence to annual albuminuria testing for eGFR <60 (defined as "Once a year" or more) |
|  |  |  |  |
|  | What clinical decision support tools do you use? | Reminder of blood pressure control; glycemic control; HbA1c order; urine testing order; physical activity; other; none | Used to assess use of urinalysis reminder system |
|  | How much do you agree or disagree with the following statements about the barriers to albuminuria testing?   - There are no guidelines for albuminuria testing or current guidelines are unclear - I have time constraints that make it difficult for me to conduct albuminuria testing - Patients often decline the urine testing - There are issues, such as no space to collect urine in the clinic or hospital, and challenges for patients to bring in or mail urine samples - The out-of-pocket cost of albuminuria testing is a concern for patients | Strongly disagree; Disagree; Neither agree nor disagree; Agree; Strongly agree | Used to assess perceived barriers: guideline ambiguity, time constraints, patient refusal, logistical issues, cost |
|  |  |  |  |

**Definitions:**

- **Outcome 1:** Adherence to quantitative albuminuria testing (Question 4: "Quantitative albuminuria")
- **Outcome 2:** Adherence to quantitative albuminuria + concurrent urine creatinine (Questions 4 & 5: "Quantitative albuminuria" and "Always" for concurrent creatinine)
- **Outcome 3:** Adherence to annual albuminuria testing for eGFR ≥60 (Questions 4 & 6: "Quantitative albuminuria" and "Once a year" or more)
- **Outcome 4:** Adherence to annual albuminuria testing for eGFR <60 (Questions 4 & 7: "Quantitative albuminuria" and "Once a year" or more)
- **Outcome 5:** Adherence to all recommended guideline components (Questions 4–7: meets all criteria above)
